# Objectively assessed long-term wearing patterns and predictors of wearing orthopaedic footwear in people with diabetes at moderate-to-high risk of foot ulceration: a 12 months observational study

**DOI:** 10.1186/s13047-023-00656-6

**Published:** 2023-09-14

**Authors:** Stein H. Exterkate, Manon Jongebloed-Westra, Peter M. ten Klooster, Hendrik Koffijberg, Christina Bode, Julia E. W. C. van Gemert-Pijnen, Josephus G. van Baal, Jaap J. van Netten

**Affiliations:** 1 Research & Development, Voetencentrum Wender, Hengelo, 7555 SK The Netherlands; 2 Department of Psychology, Health and Technology, Centre for eHealth Research and Wellbeing, University of Twente, Enschede, 7500 AE The Netherlands; 3 Department of Health Technology and Services Research, Tech Med Centre, University of Twente, Enschede, 7500 AE The Netherlands; 4 Ziekenhuisgroep Twente (ZGT), ZGT Academy, Hengelo, 7555 DL The Netherlands; 5 University of Cardiff, Cardiff, CF10 3AT UK; 6 Department of Rehabilitation Medicine, Amsterdam UMC location University of Amsterdam, Meibergdreef 9, Amsterdam, 1105 AZ the Netherlands; 7 Rehabilitation & Development, Amsterdam Movement Sciences, Amsterdam, The Netherlands

**Keywords:** Diabetes mellitus, Diabetic foot, Orthopaedic footwear, Wearing time, Treatment adherence and compliance

## Abstract

**Background:**

Orthopaedic footwear can only be effective in preventing diabetic foot ulcers if worn by the patient. Robust data on long-term wearing time of orthopaedic footwear are not available, and needed to gain more insights into wearing patterns and associated factors (i.e. participants’ demographic, disease-related characteristics, and footwear usability). We aimed to objectively assess long-term wearing patterns and identify factors associated with wearing orthopaedic footwear in people with diabetes at moderate-to-high risk of ulceration.

**Methods:**

People diagnosed with diabetes mellitus type 1 and 2 with loss of protective sensation and/or peripheral artery disease and prescribed with orthopaedic footwear were included and followed for 12 months. The primary outcome was mean daily wearing time, continuously measured using a temperature sensor inside the footwear (Orthotimer®). Adherence to wearing orthopaedic footwear was calculated as percentage of wearing time of a total assumed 16 h out-of-bed daytime, where adherence < 60% was a pre-determined non-adherent threshold. Wearing time patterns were assessed by calculating participants’ wearing (in)consistency. One-way analyses of variance tested for wearing time differences between subgroups, weekdays, and weekend days. Factors potentially associated with wearing time were collected by questionnaires and medical files. Univariately associated factors were included in multivariate linear regression analysis.

**Results:**

Sixty one participants were included (mean (SD) age: 68.0 (7.4) years; females: *n* = 17; type 2 diabetes mellitus: *n* = 54). Mean (SD) overall daily wearing time was 8.3 (6.1) hours/day. A total of 40 (66%) participants were non-adherent. Participants with a consistent wearing pattern showed higher daily wearing times than participants with an inconsistent pattern. Mean (SD) wearing times were 12.7 (4.3) vs 3.6 (4.8) hours/day, respectively (*P* < 0.001). Mean (SD) wearing time was significantly higher (*P* < 0.010) during weekdays (8.7 (6.0) hours/day) compared to Saturday (8.0 (6.1) hours/day) and Sunday (6.9 (6.2) hours/day). In the multivariate model (R^2^ = 0.28), “satisfaction with my wear of orthopaedic footwear” was positively associated (*P* < 0.001) with wearing time. The other seven multivariate model factors (four demographic variables and three footwear usability variables) were not associated with wearing time.

**Conclusions:**

Only one out of three people at moderate to high risk of foot ulceration were sufficiently adherent to wearing their orthopaedic footwear. Changing people’s wearing behaviour to a more stable pattern seems a potential avenue to improve long-term adherence to wearing orthopaedic footwear. Investigated factors are not associated with daily wearing time. Based on these factors the daily wearing time cannot be estimated in daily practice.

**Trial registration:**

Netherlands Trial Register NL7710. Registered: 6 May 2019.

## Background

In 2019 approximately 463 million adults aged 20–79 were living with diabetes mellitus [1]. These people have an increased risk of developing foot ulcers due to reducing or absence of sensory feedback, presence of peripheral artery disease and presence of foot deformities, leading to high plantar pressures [2]. When an ulcer is healed, 40% of people with diabetes develop a recurring ulcer within one year, and this increases to 60% within three years [3]. Offloading interventions including orthopaedic footwear help to reduce plantar pressure and thereby prevent plantar diabetic foot ulcer recurrence [4–6]. Adherence to wearing orthopaedic footwear is essential to prevent ulcer recurrence, but this is challenging because most patients are dissatisfied with usability of their orthopaedic shoes [7].

The first studies on footwear adherence in people with diabetes, performed in the ‘90 s and ‘00 s, showed that only 22–36% of those at risk of foot ulceration wore their prescribed footwear all day [8, 9] or at least 80% of daytime [10]. However, none of these studies were conducted in the last decade, limiting comparison to current practice that has changed with new improved footwear and new guidelines now available [11]. Furthermore, these previous studies used questionnaires or interviews to assess self-reported adherence to orthopaedic footwear, which may have low accuracy because of recall and response bias [12]. A more reliable and accurate method to objectively assess adherence is based on temperature measurements inside the footwear to identify (non-)wearing of that footwear [13]. Such an objective temperature-based sensor was used in three more recent studies on wearing diabetic footwear [14–16].

Waaijman et al. objectively measured orthopaedic shoe use in combination with daily step counts during seven consecutive days in 107 participants [14]. They showed that on average 71% of all steps were taken in orthopaedic footwear, but individual adherence rates varied widely (10 – 100%) [14]. Later, Ehrmann et al. showed a mean (standard deviation (SD)) wearing time of prescribed custom-made footwear (i.e. custom insoles in an extra-depth, stiff, rocker shoe) of 4.2 (3.6) h/day in 26 participants over a mean of 133.5 observed days [15]. Most recently, Lutjeboer et al. monitored wearing time in 11 persons with diabetes over the first 12 weeks after delivery of the orthopaedic footwear. They showed a mean wearing time of 6.95 h/day and 2.42 h/day in, respectively, the group aware of being monitored on wearing time (*n* = 6) and the no awareness group (*n* = 5) [16]. However, these studies had limitations in measurement period (seven days only in the largest study [14], 3–5 months in the smaller studies [15, 16]), and in sample size (*n* = 26 and *n* = 11 in the studies with longer follow-up [15, 16]). Robust data on longer-term wearing patterns (e.g. six months or more) of orthopaedic shoes in people with diabetes at risk of foot ulceration are still lacking. These data are necessary to gain a better insight into wearing patterns in daily practice, because we hypothesize that wearing time is not constant throughout one year follow-up.

All three previous studies showed large variations in wearing time between participants, which suggest that differences between participants might be important in adherence to wearing orthopaedic footwear. Previous studies to factors associated with adherence to wearing orthopaedic footwear had similar limitations (e.g. short measurement period, small sample sizes, self-reported adherence) and did not result in definitive conclusions [17]. As such, there is more in-depth knowledge needed about potential factors associated with adherence to wearing prescribed footwear in people with diabetes.

The aim of the current study was to objectively assess long-term wearing time, wearing patterns and identify factors associated with wearing of orthopaedic footwear (i.e. custom-made insoles in custom-made shoes) in a large group of people with diabetes at moderate-to-high risk of ulceration.

## Methods

### Study design

The cohort investigated in the current study was a control group of a 12-month cluster-randomized controlled trial (C-RCT) assessing the (cost-)effectiveness of a novel care approach (motivational interviewing) compared to usual care in improving adherence to wearing orthopaedic footwear [18]. The trial was registered in the Netherlands Trial Register, NL7710 [18] (Available on the International Clinical Trials Registry Platform). The trial was assessed as exempt from medical ethical approval by the ethical committee region Arnhem–Nijmegen, the Netherlands (NL68567.091.19) according to Dutch law, and its protocol has been published in detail elsewhere [18]. The study protocol was approved by the Ethical Committee of Behavioural, Management and Social Sciences faculty of the University of Twente (file number 190141) [18].

All participants had a temperature sensor built in their orthopaedic footwear to monitor daily wearing time (hours/day) during 12-month follow-up. The primary study outcome was mean overall daily wearing time. The secondary outcomes were wearing time patterns, assessed by calculating participants’ (in)consistency of wearing orthopaedic footwear, comparing differences between weekdays (Monday through Friday) and weekend days (Saturday and Sunday), and investigating seasonal differences. Factors potentially associated with orthopaedic footwear (i.e. participants’ demographic, disease-related characteristics, and footwear usability) were collected by questionnaires and from participants’ medical files.

### Setting

Participants were recruited at locations of Voetencentrum Wender and Voetmax Orthopedie, located in the east of The Netherlands. Eligible participants were informed about the study by the podiatrist and received an information brochure and informed consent form. After participant’s permission, the coordinating investigator contacted the participant in order to further explain the study. Thereafter, the participant had minimal one week to decide to participate. Recruitment started in July 2019 and was completed in January 2021. Participants were followed for 12 months. The orthopaedic footwear were prescribed by a medical specialist who was experienced in treating people with diabetic foot disease. Participants received usual care, as provided in standard clinical practice in the Netherlands in accordance with evidence-based guidelines [19].

### Participants

Inclusion criteria were: diagnosis of diabetes mellitus type 1 and 2 patients; age ≥ 18 years; loss of protective sensation (LOPS) and/or peripheral artery disease (PAD), and prescribed with orthopaedic footwear for foot deformities (International Working Group on the Diabetic Foot (IWGDF) risk 2–3) [11]. All participants were screened for eligibility by trained podiatrists. LOPS was measured using the 10 g Semmes–Weinstein monofilament [20] and PAD using an audible handheld Doppler (Huntley Digital Doppler®; Huntleigh Healthcare Ltd, Cardiff, Wales), with the diagnosis based on presence or absence of triphasic pedal Doppler waveforms [21]. Exclusion criteria were: inability to follow study instructions; active Charcot’s neuro-arthropathy; foot infection; or being unable to walk. Written informed consent was obtained from each participant prior to inclusion in the trial.

On the informed consent form, participants agreed to the sensor placement and data storage. In both the information brochure and informed consent form participants were not notified that the sensor was used to monitor daily wearing time; it was only described as temperature monitoring sensor. Logged temperature data were collected from the microsensors every three months. These moments were mostly combined with regular appointments with a pedorthist or podiatrist. Otherwise data were read out during an additional appointment or at the participant’s home. Participants who withdrew or were deceased before the first sensor reading were excluded from further analysis. Drop-outs after the three-month mark were included in the analysis, including reason registration for withdrawn.

Measuring days from periods in which participants (re-)experienced complications (e.g. diabetic foot ulcer, lower-extremity amputation, or hospitalization) that could have affected wearing time were excluded from analysis. These complication periods were selected by retrospectively screening participants’ medical files after study completion. Whenever either the start or end date of a complication period was unknown, an exclusion period of 165 days was used based on diabetic foot ulcer (DFU) healing time showed in a recent study conducted in the same geographical region [22].

### Instrumentation

Every pair of orthopaedic footwear that participants possessed and used at study entry (i.e. earlier prescriptions) or that was prescribed and provided during follow-up was included in the study and equipped with a microsensor (Orthotimer®; Rollerwerk medical engineering & consulting, Balingen, Germany). The sensor was placed in the medial arch of the shoe insole because of sufficient place in the insole, relatively low pressure from the foot, and its previous validation at this location [23]. The sensor stored temperature with a date- and timestamp every 20 min and had a storage capacity of 133 days before overwriting the oldest data. At 12 months, participants were asked to fill in the Monitor Orthopaedic Shoes (MOS) questionnaire to measure their perception regarding their orthopaedic footwear use and usability, and their subjective assessment of their wearing behaviour [24].

### Variables

#### Wearing time

The total daily wearing time of all pairs of orthopaedic footwear during the 12-month follow-up was based on logged temperature data with date- and timestamps from the sensors, and calculated with the validated Groningen algorithm, version 2, using Matlab (R2017a, The MathWorks, Inc., Natick, Massachusetts, United States) [23, 25]. The primary outcome was the participants’ mean overall daily wearing time (hours/day) during the study, and was calculated as:

$$mean\,daily\,wearing\,time= \frac{\sum_{i=1}^{n_{days}}\sum_{i=1}^{n_{sensors}}daily\,wearing\,time(\frac{hours}{day})}{{n}_{days}}$$

Besides wearing time, adherence to wearing orthopaedic footwear was calculated as percentage of wearing time of a total assumed 16 h out-of-bed daytime, to compare outcomes with previous studies using the same adherence definition (adherent ≥ 80%, medium adherent ≥ 60% < 80%, non-adherent < 60%) [10, 14, 26]. Missing data (i.e. due to delayed sensor readings or drop-outs after three-months) or invalid data (i.e. summed daily wearing time ≥ 24 h or measuring days from periods in which participants (re-)experienced complications) were not imputed.

#### Wearing time patterns

Secondary outcomes were the wearing time patterns and factors potentially associated with wearing time. Patterns based on (in)consistency of wearing orthopaedic footwear were assessed by calculating the coefficient of variation (CV) for each participant over the 12-month follow-up, defined as the ratio of the standard deviation to the mean wearing time [27]. The CV is a standardized measure of dispersion. Participants were split into tertiles from low to high CV. Participants in the low CV tertile had the most consistent wearing pattern and those in the high CV tertile had the most inconsistent wearing pattern. To assess seasonal differences in wearing time, astronomical seasonal periods were used; Spring (21^st^ of March – 20^th^ of June), Summer (21^st^ of June – 20^th^ September), Autumn (21^st^ of September – 20^th^ of December), and Winter (21^st^ of December – 20^th^ of March). Participants were included in the comparison of seasonal wearing times when at least 50% of seasonal days were assigned as valid during each season.

#### Predictors

Demographic data (i.e. gender, age, body mass index (BMI), education level, working situation, living situation, self-reliance, dependence on an assistive device) and disease-related characteristics (i.e. diabetes type, diabetes duration, IWGDF risk profile) were collected using participants’ medical files and self-report at study entry. Footwear usability variables (i.e. walking ability, perceived walking change by orthopaedic footwear, shoe fit, shoe walking, shoe weight, donning and doffing, aesthetic, aesthetic perceived by others, number of orthopaedic footwear pairs, footwear possession, owns regular off-the-shelf shoes, satisfaction with my wear of orthopaedic footwear, orthopaedic footwear wearing goal reached) were collected using the MOS-questionnaire at 12 months.

### Statistical analyses

Statistical analysis was performed using SPSS statistical software (V.28.0, SPSS, New York, USA), with significance level of *p* < 0.05. Wearing time was stated to fit a normal distribution (Anderson–Darling test; *p* = 0.368). Descriptive statistics for wearing time were calculated as the mean (SD) for all participants, wearing (in)consistency subgroups (low CV, medium CV, and high CV), adherent subgroups (non-adherent, medium adherent, adherent), weekdays, and weekend days.

One-way analyses of variance (ANOVA) tested for differences between (in)consistency subgroups, adherent subgroups, week and weekend days, and seasonal periods. Tukey–Kramer post-hoc analyses were applied for pairwise comparisons. Univariate linear regression tested the associations with the dependent variable daily wearing time for all dichotomous and continuous independent variables. Variables with *p* < 0.20 were entered into a forward multivariate linear regression analysis to identify unique determinants of wearing time. Collinearity between independent variables was tested by linear regression, where Pearson’s correlation coefficients ≥ 0.70 were defined as correlated. In the event of collinearity where both variables also had a near significant (*p* < 0.20) correlation with wearing time, only the variable with highest association with daily wearing time was entered in the multivariate linear regression model. Post-hoc power analyses based on a two-sided alpha of 0.05 and power of 0.80 were performed (version 3.1.9.7, G*Power, Germany) to test whether the sample size met for subgroups comparisons and multivariate linear regression analysis.

## Results

**Fig. 1:**
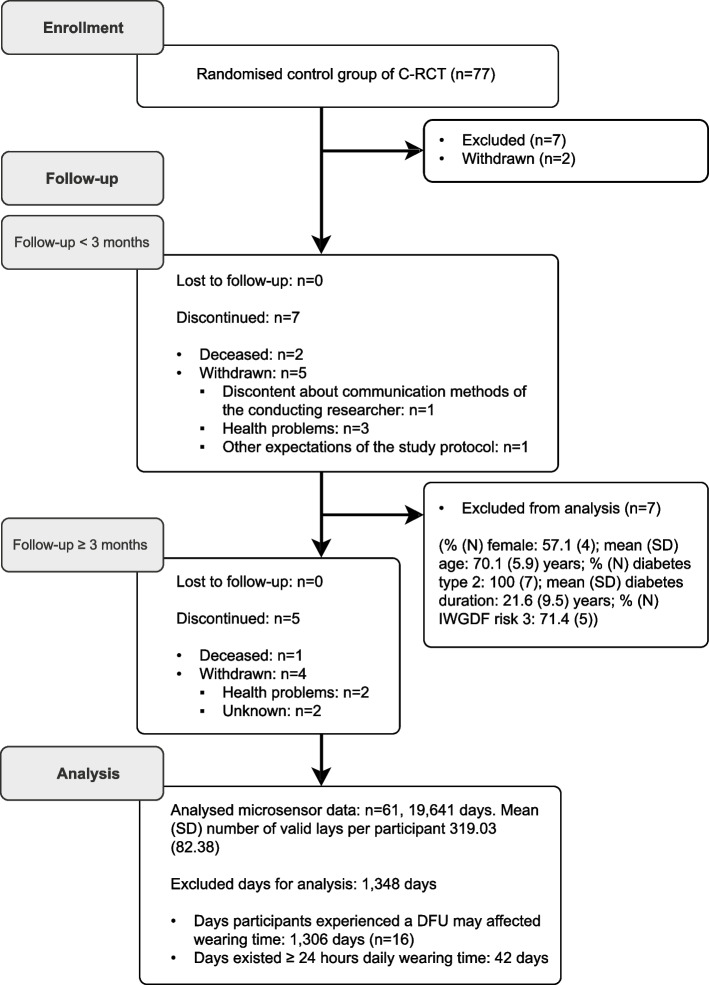
Flowchart of participants included in this study. Abbreviations: IWGDF: international working group on the diabetic foot, SD: standard deviation, DFU: diabetic foot ulcer, C-RCT: cluster-randomized controlled trial

A study flowchart is shown in Fig. 1, and a summary of the participants’ data is shown in is Table 1.

**Table 1:** Descriptive statistics, univariate regression, and multivariate regression of investigated variables in relation to daily wearing time

Characteristic	Mean (SD)	% (N)	Wearing time Mean (SD)	Univariate regression			Multivariate regression	
				B	β	*p*-value	β	*p*-value
**Demographics**								
**Gender**								
Male		72 (44)	7.9 (5.9)					
Female		28 (17)	9.4 (6.2)	2.03	0.21	0.100^a^	0.12	0.356
**Age (years)**	68.0 (7.4)	100 (61)	8.3 (6.1)	0.13	0.22	0.083^a^	0.13	0.322
**BMI**	30.5 (5.7)	100 (61)	8.3 (6.1)	-0.14	-0.19	0.145^a^	-0.15	0.242
**Education level**								
Low		49 (30)	9.6 (5.8)					
Medium/High		51 (31)	7.1 (6.0)	-2.95	-0.34	0.007^a^	-0.19	0.138
**Working situation**								
Paid work		28 (17)	7.8 (6.3)					
No paid work		72 (44)	8.5 (6.0)	0.88	0.09	0.480		
**Living situation**								
Living with someone		71 (43)	8.2 (6.1)					
Living alone		30 (18)	8.5 (5.8)	0.11	0.01	0.926		
**Self-reliant**								
Yes		16 (10)	8.3 (5.6)					
No		84 (51)	8.3 (6.1)	-0.37	-0.03	0.809		
**Dependence on assistive device**								
Yes		34 (21)	8.2 (6.1)					
No		66 (40)	8.4 (6.0)	0.40	0.04	0.735		
**Disease characteristics**								
**Diabetes type**								
Type 1		11 (7)	7.8 (6.3)					
Type 2		89 (54)	8.4 (6.0)	0.44	0.03	0.801		
**Diabetes duration (years)**	17.3 (11.4)			0.05	0.14	0.303		
**IWGDF risk profile**								
IWGDF risk 2		44 (27)	8.5 (6.1)					
IWGDF risk 3		56 (34)	8.1 (6.0)	-0.82	-0.10	0.465		
**Footwear usability**								
**Walking ability**								
< 1000 m		70 (35)	8.1 (6.0)					
≥ 1000 m		30 (15)	8.8 (6.3)	0.41	0.04	0.762		
**Perceived walking change by OF**								
Improved by orthopaedic footwear		52 (26)	9.2 (5.7)					
Not improved by orthopaedic footwear		48 (24)	7.2 (6.4)	-1.56	-0.18	0.207		
**Shoe fit**^c^	80.7 (18.4)	82 (50)	8.3 (6.1)	0.03	0.11	0.461		
**Shoe walking**^c^	78.6 (24.5)	80 (49)	8.3 (6.1)	0.04	0.24	0.093^a^	-0.18	0.283
**Shoe weight**^c^	56.5 (22.0)	79 (48)	8.3 (6.1)	-0.05	-0.25	0.086^a^	-0.02	0.884
**Donning and doffing**^c^	68.0 (26.3)	79 (48)	8.4 (6.1)	0.005	0.03	0.852		
**Aesthetic**^c^	75.9 (21.0)	80 (49)	8.3 (6.1)	< 0.001	0.01	0.975		
**Aesthetic perceived by others**		84 (51)						
Not attractive		43 (22)	7.8 (5.8)					
Attractive		57 (29)	8.7 (6.3)	0.80	0.09	0.514		
**Number of orthopaedic footwear pairs**	2.9 (1.1)	100 (61)	8.3 (6.1)	0.65	0.17	0.204		
**Footwear possession**								
First-ever pair		13.1 (8)	9.3 (5.8)					
Subsequent pair		86.9 (53)	8.2 (6.1)	-0.47	-0.04	0.776		
**Owns regular off-the-shelf shoes**								
Yes		20 (12)	6.2 (5.6)					
No		80 (49)	8.9 (6.0)	2.34	0.22	0.093^a^	0.15	0.238
**Satisfaction with my wear of orthopaedic footwear**^c^	80.1 (20.9)	82 (50)	8.3 (6.1)	0.11	0.52	< 0.001^a^	0.55	< 0.001^b^
**Orthopaedic footwear wearing goal reached**								
Yes		81 (39)	8.8 (6.1)					
No		19 (9)	6.1 (5.6)	-1.85	-0.17	0.264		

Percentages may not added up to 100 due to rounding

*Abbreviations: SD* standard deviation, *B* unstandardized coefficients, *β* standardized coefficients, *BMI* body mass index, *IWGDF* International working group on the diabetic foot

^a^Variables with *p*-values < 0.20 in the univariate regression were entered in the multivariate regression model

^b^*p* < 0.05 in the multivariate regression analysis. Multivariate regression model F_(1,44)_ = 18.64, *p* < 0.001, *R*^*2*^ = 0.28

^c^Scores could range from 0 (lowest/most negative score possible) to 100 (highest/most positive score possible)

### Wearing time

Over the total group of participants (*n* = 61), mean (SD) wearing time was 8.3 (6.1) hours/day (Table 2). A total of 34% (*n* = 21) were adherent (≥ 60% of out-of-bed daytime), while 66% (*n* = 40) were non-adherent (< 60% of out-of-bed daytime).

**Table 2:** Daily wearing time (hours/day) for all days, Saturday, Sunday, and weekdays per subgroup

Subgroup	% (N)	Full measurement period	Weekdays^k^	Saturday	Sunday
Total	100 (61)	8.3 (6.1)	8.7 (6.0)^gi^	8.0 (6.1)^di^	6.9 (6.2)^cf^
Non-adherent (< 60%)	66 (40)	5.8 (5.3)^a^	6.2 (5.3)^fi^	5.4 (5.3)^ci^	4.3 (5.0)^cf^
Medium adherent (≥ 60 < 80%)	16 (10)	11.4 (4.8)^a^	12.0 (4.6)	11.0 (4.2)	9.1 (5.7)
Adherent (≥ 80%)	18 (11)	14.7 (3.1)^a^	14.8 (3.1)	14.7 (3.1)	14.3 (2.9)
CV_low_	33 (20)	12.7 (4.3)^b^	13.0 (4.2)	12.3 (4.2)	11.6 (4.8)
CV_mid_	34 (21)	8.0 (5.3)^b^	8.4 (5.2)^j^	8.2 (5.4)^j^	6.1 (5.4)^dg^
CV_high_	33 (20)	3.6 (4.8)^b^	4.0 (4.9)^hj^	2.8 (4.5)^e^	2.3 (4.4)^d^

Data are expressed as mean (standard deviation)

*Abbreviations:* *CV* coefficient of variation. CV tertile cut-off levels: CV_low_ ≤ 0.45, CV_high_ > 0.81

^a^*P* < 0.01 significantly differences between adherent subgroups

^b^*P* < 0.001 significantly differences between CV tertiles

^c^*P* < 0.001 significantly different from weekdays

^d^*P* < 0.01 significantly different from weekdays

^e^*P* < 0.05 significantly different from weekdays

^f^*P* < 0.001 significantly different from Saturday

^g^*P* < 0.01 significantly different from Saturday

^h^*P* < 0.05 significantly different from Saturday

^i^*P* < 0.001 significantly different from Sunday

^j^*P* < 0.01 significantly different from Sunday

^k^Weekdays: Monday through Friday

### Wearing time patterns

**Fig. 2:**
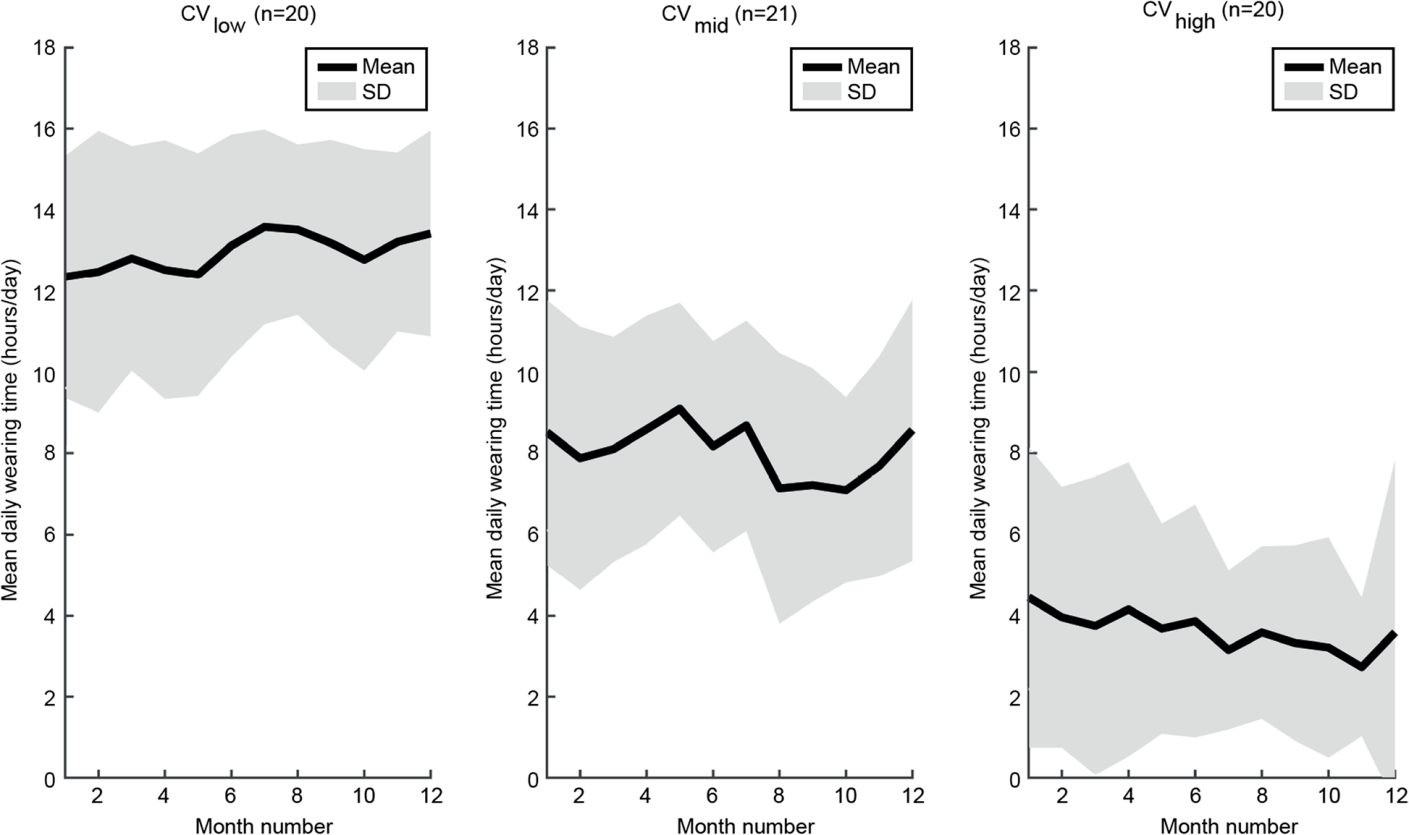
Daily wearing time over one year follow-up for participants split into CV tertiles. Abbreviations: CV: coefficient of variation, SD: standard deviation. Cut-off levels: CV_low_ ≤ 0.45, CV_high_ > 0.81

Wearing time was higher during weekdays compared to Saturday and Sunday (*p* < 0.010; Table 2). This pattern was the same for all subgroups, but the difference was not always statistically significant in the subgroups (Table 2). Participants in the smallest CV tertile (i.e. most consistent wearing time during 12 months) showed the highest wearing time, while those in the largest CV tertile (i.e. most inconsistent wearing pattern) showed the lowest (*p* < 0.001; Fig. 2; Table 2). Seasonal differences between mean (SD) daily wearing time were small (Spring: 8.2 (6.0), Summer: 8.4 (6.1), Autumn: 8.0 (6.0), and Winter: 8.5 (6.2) hours/day) and non-significant (*p* = 0.312).

### Predictors

Univariate analyses of participant demographics showed higher wearing times for female participants, older participants, participants with a lower BMI, and those with a lower educational level (*p* < 0.20; Table 1). Four variables of footwear usability showed a univariate association with wearing time (*p* < 0.20; Table 1). No variables associated with wearing time showed any collinearity. In the multivariate regression model, the variable “satisfaction with my wear of orthopaedic footwear” remained significantly positively associated (*p* < 0.001; Table 1) with wearing time. The model consisted of eight variables (four demographic variables and four footwear usability variables) and explained 28% of the variance in wearing time.

### Post-hoc power calculations

Post-hoc power sensitivity analyses indicated that this study had sufficient power (80%) to significantly (*p* < 0.05) detect large between-group differences (F = 0.41 – 0.44) for 3 to 4 subgroups. For multivariate linear regression analysis with 8 potential predictors and 80% power, a medium to large proportion of variance could be explained (*F*^*2*^ = 0.28; R^2^ = 0.22).

## Discussion

The aim of the study was to investigate objectively measured long-term wearing time of orthopaedic footwear, wearing patterns, and identify factors associated with wearing in people with diabetes at moderate-to-high risk of ulceration. A wide range in daily wearing time was found, indicating large differences between participants. The mean daily wearing time was 8.3 h, which we consider low given an average 16 h daily out of bed time. Wearing times were higher during weekdays compared to Saturday and Sunday, with Sunday also less than Saturday. Participants with a stable wearing pattern (i.e. a low CV) showed on average higher daily wearing time than participants with more fluctuations in their wearing pattern (i.e. a high CV). Seasonal differences between wearing time were negligible. Of all demographics, disease-related characteristics, and footwear usability variables, only “satisfaction with my wear of orthopaedic footwear” was statistically significantly associated with daily wearing time in multivariate analysis.

Our study shows similar daily wearing time compared to two quantitative studies (9.4 ± 4.4 and 7.0 ± 4.7 h/day) [14, 16], whereas compared to third quantitative study available (4.2 ± 3.6 h/day) the current study shows higher wearing time [15]. All studies on this topic to date show low wearing times and large differences between participants. This supports the idea that reasons for wearing orthopaedic footwear is an individual matter and should be improved. In this study, 66% of participants wore their orthopaedic footwear < 60% of daily out of bed time, where ≥ 60% was thought to reduce the rate of ulceration [28]. One quantitative [14] and two qualitative studies [10, 26] showed respectively 33% and 58% of participants with wearing times < 60% of daily out of bed time,. The selection criteria in the previous quantitative study [14] (i.e. history of a recent plantar DFU) may partly explain the difference with the current study result, as we also included participants without an ulcer history, or with an ulcer longer ago and therefore were at lower risk of developing a diabetic foot ulcer [11]. However, adherence to wearing orthopaedic footwear is suboptimal in most participants and must improve to prevent diabetic foot ulcers.

We found higher wearing times during weekdays compared to weekend days, similar to a previous quantitative study [14]. This effect was largest for subgroups with the lowest wearing times. Participants and clinicians should be aware of the importance to wear orthopaedic footwear every day, also – or especially – during weekend days. A new finding in this study concerned the (in)consistency in wearing patterns, where participants with a consistent wearing pattern (CV_low_) showed significantly higher daily wearing times than participants with an inconsistent pattern. This suggests that a stable wearing pattern is mostly associated with high daily wearing time, those participants likely formed habits to often wear their orthopaedic footwear. This is supported by a recent qualitative study that showed that consistent choices about which footwear type to wear was positively associated with adherence to wearing therapeutic footwear [29]. Therefore, changing patients’ wearing behaviour to a more stable pattern may be a potential avenue to improve long-term adherence to wearing orthopaedic footwear.

The multivariate model explained 28% of the wearing time variance, and showed that “satisfaction with my wear of orthopaedic footwear” was positively significantly associated with wearing time. The model showed that a low education level was associated with higher wearing time, although not significantly. This was unexpected and the reason remains unclear. Previous studies did not found any impact from education level on adherence [14, 29]. Despite, the explained variance was higher compared to multivariate models in previous studies (6–18%) containing similar variables [14, 29], there was still a substantial amount of unexplained variance. Both quantitative and qualitative studies were previously conducted to investigate similar factors associated with adherence to wearing footwear, showing both supportive and contradictory results [10, 14, 26, 29, 30]. Combining the results from these multiple studies, it seems that demographics, disease-related characteristics, and footwear usability variables are not useful for predicting orthopaedic footwear wearing time in people with diabetes. Patients’ adherence to wearing orthopaedic footwear cannot be estimated by clinicians based on these factors. Other previous studies showed that someone’s decision to use orthopaedic footwear can be influenced by the communication style of the healthcare provider, which is associated with increased long-term footwear usefootwear [7, 31]. However, adequately powered randomized controlled trials are needed to establish the efficacy of communication styles in improving adherence to wearing orthopaedic shoes [32–34]. Therefore, to determine patients’ adherence to wearing orthopaedic footwear in daily practice it should be objectively measured on an individual level rather than estimated.

### Limitations

The results of this study may be limited by the following: firstly, recruitment took place during the Covid-19 pandemic (July 2019 – January 2021). During this period people were recommended to work from home or not to work at all. Because of this, participants have likely spent more time at home than usual. This may have influenced wearing times, since wearing time is often higher away from home than at home [14].

Secondly, participants were asked to bring every pair of orthopaedic footwear they already possessed at study entry to the first study appointment, so all these footwear could be equipped with a sensor. However, during the study it was found that some participants had more orthopaedic footwear than they brought during the first appointment. This may have resulted in an underestimation of wearing time.

Thirdly, participants were not notified that the sensor was used to monitor daily wearing time. This is in line with the information given by the researcher on an unaware group in a previous study showing a positive effect of awareness of being monitored on wearing orthopaedic footwear [16]. As such, we consider that participants could be regarded as being unaware. We did not assess at the end of the study whether the participants believed this or not, and whether this affected wearing times.

Finally, it should be noted that with 61 participants in the current study, this study lacked statistical power to detect small differences between subgroups or to detect independent factors that may be predictive of wearing time as statistically significant. However, the current study results are in line with a previous study with a larger sample size that fail to detect strong associations with wearing time for similar variables [29].

### Future research

First, inconsistent long-term wearing patterns were seen in participants with low daily wearing time. Changing wearing time to a more consistent pattern may result in new habits that contribute to higher long-term wearing times [29]. Therefore, future research should explore strategies to change wearing behaviour to a stable pattern. Clinicians can discuss these strategies with patients to form new footwear habits, so wearing orthopaedic footwear become the default option without conscious effort.

Secondly, since adherence to wearing orthopaedic footwear cannot be explained by investigated factors, we recommend that the communication style of the healthcare provider, and the influence of other factors like individual patients’ perspective with regard to their orthopaedic footwear should be investigated. Moreover, it is known that patients have different perceptions with regard to what characteristics of orthopaedic footwear are important to them [26, 31, 35]. Mixed-method research combining objectively measured wearing time with qualitative components through triangulation is needed to obtain the effect of patients’ perspective on their orthopaedic footwear to daily wearing time. Thereafter, these individual perspectives might be used in questionnaires to assess patients orthopaedic footwear use and usability in daily practice.

## Conclusion

Only one out of three people with diabetes at moderate-to-high risk of foot ulceration were sufficiently adherent to wearing their orthopaedic footwear during 12 months.

People with a consistent wearing pattern show higher daily wearing times than people with an inconsistent pattern. Further, people wear their orthopaedic footwear less during weekend days compared to weekdays. By changing wearing behaviour to a more stable pattern seems a potential avenue to improve long-term adherence to wearing orthopaedic footwear.

Only self-reported “satisfaction with my wear of orthopaedic footwear” is positively associated with wearing time. All other investigated factors are not associated with wearing time. Based on these factors patients’ daily wearing time cannot be estimated in daily practice.

## Data Availability

The datasets used and/or analysed during the current study are available from the corresponding author on reasonable request after publication of the results.

## Abbreviations

ZGT: Ziekenhuisgroep Twente
SD: Standard deviation
C-RCT: Cluster-randomized controlled trial
LOPS: Loss of protective sensation
PAD: Peripheral artery disease
IWGDF: International Working Group on the Diabetic Foot
DFU: Diabetic foot ulcer
MOS: Monitor Orthopaedic Shoes
CV: Coefficient of variation
ANOVA: One-way analyses of variance
BMI: Body mass index
WMO: Medical research involving human subjects act; in Dutch: Wet medisch-wetenschappelijk onderzoek met mensen
